# Fungal Infections among Teledermatology Consultations in Dermatology Department of a Tertiary Care Hospital: A Descriptive Cross-sectional Study

**DOI:** 10.31729/jnma.5900

**Published:** 2021-11-30

**Authors:** Sagar Mani Jha, Anil Kumar Singh Dangol, Bhabendra Suwal, Jyotshna Yadav

**Affiliations:** 1 Department of Dermatology, Nepalese Army Institute of Health Sciences, Kathmandu, Nepal

**Keywords:** *communication*, *technology*, *teledermatology*

## Abstract

**Introduction:**

Teledermatology provides virtual consultation to patients using telecommunication technology. Using this method dermatologists can diagnose a condition with the help of pictures of the lesions and short history. During the COVID-19 pandemic, practicing this method has become more relevant. Providing teleconsultations to patients with fungal skin infections can prevent inadvertent use of topical corticosteroids. The objective of this study was to find the prevalence of fungal infections among teledermatology consultations done in a tertiary care hospital.

**Methods:**

It was a descriptive cross-sectional study where the store and forward and real-time methods were used between February 2020 to July 2020. Ethical clearance was taken from the institutional review board (reference number: 245). Data of those patients who wanted consultations from this department were sent by medical officers deployed in military hospitals that are under the central army hospital located in Kathmandu. Convenient sampling was used. The collected data was entered and analyzed in the Statistical Package of Social Sciences version 20. Point estimate at 95% Confidence Interval was calculated along with frequency and percentage for binary data.

**Results:**

A total of 451 (33.45%) (30.93-35.97 at 95% Confidence Interval) were diagnosed with fungal infections out of 1348 patients who were enrolled for the study. About 361 (80%) of the patients suffering from fungal infections belonged to the Terai region and 90 (20%) belonged to mountainous areas.

**Conclusions:**

The prevalence of fungal infection among teledermatology consultation was lower than the findings from a similar international study.

## INTRODUCTION

Superficial fungal infections can easily be diagnosed and treated with the help of teledermatology. Teledermatology is a method of providing services in difficult terrain and rural areas where healthcare services are scarce. With the introduction of newer technologies like third-generation (3G) and fourth-generation (4G), dermatologists are adopting the practice to give an expert opinion to address the cases which have resulted in a rapid change in teledermatology practice.^1^

Fungal infections are prevalent all over the country with Tinea infections much common among out-patients. Patients often go to pharmacies for the treatment and are frequently given topical steroids leading to further spread and side effects. Teledermatology could help reduce treatment cost, difficulty of movement and would give these patients opportunity to have fast service during lockdown. The goal is to provide the highest quality of dermatologic care more efficiently by moving patient information rather than the patient.^2^

The aim of this study was to find out the prevalence of fungal infections among teledermatology consultations in a tertiary care hospital.

## METHODS

It was a descriptive cross sectional based study where mainly store and forward (SAF) and real-time methods (RTM) were used. The medical officers deployed in various military hospitals under the central army hospital (Shree Birendra Hospital) located in Kathmandu, Nepal took photographs and relevant data and sent them to the Department of Dermatology for a period of six months between February 2020 to July 2020. Ethical clearance was taken from the Institutional Review Board of Nepal Army Institute of Health Sciences (Reference no.245). Medical officers chose only those patients who were willing to seek consultation from this department of dermatology and photos of only those patients were sent for consultation after the written consent about their participation and sending of pictures was obtained and those patients who didn't come for a consultation with this department were excluded from the study. Patients were fully explained about the whole procedure which included taking photographs, history of illness, sending all the data to the central hospital, and receiving dermatological advice. Only new patients who visited for the first time were included and those who were already seeking consultation before the commencement of the study were excluded from the study. Convenience sampling was done and the sample size was calculated using the formula,

n = Z^2^ × p × q / e^2^

  = (1.96)^2^ × 0.5 × (0.5) / (0.04)^2^

  = 600

Where,

n= required sample size,
Z= 1.96 at 95% Confidence Interval (CI),
p= prevalence taken as 50% for maximum sample size,
q= 1-p
e= margin of error, 4%

Since we used convenience sampling to enroll participants, we doubled the sample size to 1200 to make the sample as closely representative of the target population as possible. Adding a 10% non-response rate, the final sample size became 1320. However, we collected data from 1348 participants.

Third generation (3G) and fourth-generation(4G) mobile technology was used to transfer data. These photographs along with short history were analyzed in the department by a panel of dermatologists. Photos were taken from various apparatus including smartphones and images were stored in JPEG format. Time was fixed for the real-time session with the doctors who had sent photos. Each case was discussed and additional information was taken as and when required. The diagnosis and treatment were sent to referring doctors electronically using emails or mobile messaging applications.

The data collected during the study were tabulated, coded and analyzed using the Statistical Package for the Social Sciences (SPSS) version 20 and variables were expressed as frequencies and percentages. Point estimate at 95% CI was calculated along with frequency and percentage for binary data.

## RESULTS

Out of 1348 patients who were enrolled in the study, a total of 451 (33.45%) (30.93-35.97 at 95% CI) were diagnosed with fungal infections. Age ranged between 6 months to 86 years and males were 290 (64.3%) and females were 161 (35.7%).

About 361 (80%) of the patients suffering from fungal infections belonged to the Terai region and 90 (20%) belonged to mountainous areas. Tinea Cruris 170 (37.7%) and Pityriasis Versicolor 135 (30%) was most prevalent in terai region whereas tinea capitis 24 (5.3%) was more prevalent in mountainous region. People between 20 to 45 years of age suffered most with this infection with the mean age of 34 years. The number of same people suffering from fungal infections at the various sites of their body were 193 (42.7%). The percentage of people suffering from tinea corporis was comparable in both the regions. Patients who took teledermatology consultations also suffered from Acne vulgaris, Hand eczema, Verruca Vulgaris, and other wide range of diseases. Among all patients diagnosis could not be made in 94 (6.97%).

**Figure 1:**
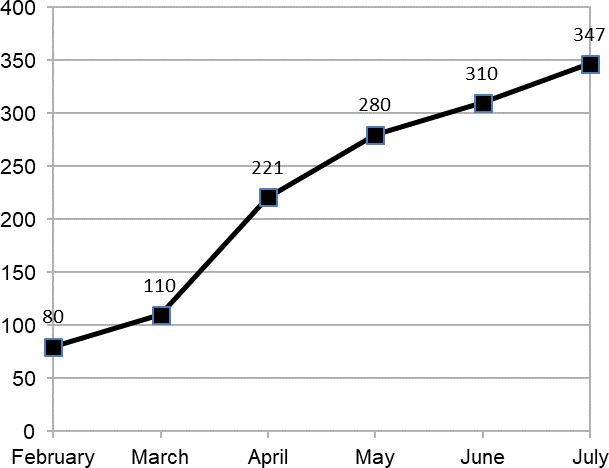
Graph depicting the month-wise increase in the number of patients.

It was also found that the number of patients taking consultation increased from 80 in February 2020 to 347 in July 2020. There was an increase of 30 patients taking consultation from February to March whereas there was an increase of 111 patients after lockdown due to COVID-19 from March to April (Figure 1).

## DISCUSSION

Telemedicine is defined as the use of technologies and their applications of telecommunication to provide medical information and services.^3^ The application of the principles of telemedicine to dermatology is generally referred to as teledermatology.^4^ Some centers conduct proper teledermatology with proper recommended equipment and base in rural areas. Communicationtechnology has reached almost all the districts of Nepal. The store and forward (SAF) method is widely used mainly due to poor connectivity in using the real time method to send data and receive an expert opinion. The introduction of newer technologies like third-generation (3G) and fourth-generation (4G) mobile teledermatology and the interest of dermatologists to adopt teledermatology practice to give an expert opinion to address the cases has resulted in a rapid change in teledermatology practice.^1^

Nepal has difficult terrain and dermatologists are not available everywhere so the use of teledermatology is very useful for patients who don't have access to dermatologists as and when required. Moreover, over the counter use of topical steroids has made the matter worse. With the use of teledermatology, patients from remote areas are getting services as telecommunication is widely available. Without using expensive equipment photographs and short history are received using mobile phones. In this study, there was a significant increase in the number of patients after lockdown imposed due to COVID-19 at the end of March from 110 patients to at the end of April 221 patients. In the present-day context during the COVID-19 pandemic, this method of teleconsultation has become more relevant as dermatology is mainly a visual specialty,teledermatology can be an important tool in reaching out to patients, especially in times of a pandemic, to reduce patient load and overcrowding in outpatient departments.^6^

In this study the maximum number of patients were suffering from fungal infections which included Tinea Corporis, Tinea Cruris, Tinea Capitis, and Pityriasis Versicolor. In the normal out patient department of this hospital the 16% of the patients who took direct consultation from dermatologists had fungal infections as compared to our teledermatology study where 33.45% were suffering with the same infections.^5^ In a teledermatology study conducted at Jammu in India by Gupta M and Bhargava S showed the prevalence of fungal infection to be 15.7%.^6^ The high prevalence of infections in our study could be attributed to the paucity of dermatology services in Nepal. Similar to our observation, in a study conducted by Kaliyadan F and Venkitakrishnan S diagnosis of fungal infections were made with more certainty.^3^ Among all fungal infections 361 (80%) patients belonged to the Terai region of Nepal. The number of patients suffering from other conditions like scabies, Impetigo, and Psoriasis was comparable between people living in the terai and mountainous regions.

Treatment was started in all patients and were asked to follow up in one month. Patients who came for follow-up had either significant improvement in the lesions or the lesions had completely resolved. It was easy to follow up with patients because their patient cards and contact details were maintained at the local medical units. In this study in 6.97% of cases, diagnosis could not be made as compared to 4.2% in a study conducted by Okay and Rennie in New Zealand in Rotorua and Taumarunui.^7^ We recommended direct consultation of these patients with dermatologists for further investigation and management.

To make a diagnosis based on photograph and short history the camera used must have good resolution and secondly the person who is taking photographs must have at least minimum knowledge of dermatology. The diagnosis is mainly based on the quality of images submitted for consultation and it becomes more important especially if the referring physician is not familiar with dermatological photography.^8^ Ideally, the images and patient details can be sent initially for the specialist's preview and then a time can be set-up for a video teleconferencing during which the specialist can fill any information gaps or focus on any other area of interest on the skin, as required.^3^ To make things simple and in order to reach more and more patients the whole procedure was simplified by using the photos taken with the help of mobile phones. At times it was noted that photographs were not up to the standard but complementing the photo with history and symptoms in direct interview the diagnosis could be made with high accuracy.

Medico legal issues must be taken into consideration. Privacy legislation in Australia has made access to the blogs possible, only by invitation.^9^ Prevailing laws about telemedicine and teledermatology has to be abided. Ethical issues and privacy of the patients must be ensured during the whole process of teledermatology.

Confidentiality and security features are of paramount importance to any teledermatology process, and records must be linked to patients' regular health cards for maximum effectiveness.^10^ In a resource poor setup cost is a major factor and it determines the efficiency and smooth conduction of whole service however cost-effectiveness studies have been limited by addressing only a few economic principles.^11^ Teledermatology practice reduces frequent visits to the doctors, travel time and cost, waiting period of the patients and minimizes the overall treatment cost.^12^

## CONCLUSIONS

The prevalence of fungal infection among teledermatology consultation was lower than the findings from a similar international study and patients seeking teledermatology services increased during COVID-19 lockdown. Teledermatology is emerging at a rapid pace in Nepal because not all patients have direct access to dermatologists as and when required. Simple mobile phone with a camera is very handy and easy to use by medical officers and general practitioners to send data to dermatologists. Patients can be benefitted without physically visiting the clinic. In the present day context with COVID-19 pandemic teledermatology has become the preferred method for consultation with dermatologists.
